# Correlation between the chemical composition and the antimicrobial properties of seven samples of essential oils of endemic Thymes in Morocco against multi-resistant bacteria and pathogenic fungi

**DOI:** 10.1016/j.jsps.2022.06.022

**Published:** 2022-06-22

**Authors:** Aziz Drioiche, Fatima Zahra Radi, Atika Ailli, Amal Bouzoubaa, Amale Boutakiout, Soumia Mekdad, Omkulthom AL Kamaly, Asmaa Saleh, Mohamed Maouloua, Dalila Bousta, Server Sahpaz, Fadoua EL Makhoukhi, Touriya Zair

**Affiliations:** a Research Team of Chemistry of Bioactive Molecules and the Environment, Laboratory of Innovative Materials and Biotechnology of Natural Resources, Faculty of Sciences, Moulay Ismaïl University, B.P. 11201 Zitoune, Meknes 50070, Morocco; b Medical Microbiology Laboratory, Mohamed V. Hospital, Meknes, Morocco; c Department of Pharmaceutical Sciences, College of Pharmacy, Princess Nourah bint Abdulrahman University, P.O. Box 84428, Riyadh 11671, Saudi Arabia; d Laboratory of Biotechnology, Health, Agrofood and environment (LBEAS), Faculty of Sciences Dhar El Mehraz, Sidi Mohamed Ben Abdellah University, Fez 30000, Morocco; e Univ. Lille, University of Liège, University of Picardie Jules Verne, JUNIA, UMRT 1158 BioEcoAgro, Specialized Metabolites of Plant Origin, F-59000 Lille, France

**Keywords:** Thymus vulgaris, Thymus satureioides, Thymus zygis, Carvacrol, Thymol, Borneol, Multiresistant microorganisms, MIC, MBC, MFC

## Abstract

Thymus vulgaris, Thymus satureioides, and Thymus zygis are endemic Moroccan species that are intensively used due to their extensive medications and culinary properties. To enhance and preserve these overexploited species, the effect of provenance on the chemical composition of essential oils and antimicrobial activity against human pathogens were studied. Essential oils (EO) obtained by hydrodistillation from the flowering tops of thyme species were analyzed by GC-SM. The determination of minimum inhibitory (MIC), bactericidal (MBC), and fungicide (MFC) concentrations of EO were studied by microplate microdilution. The correlation between the chemical composition of EO and antimicrobial properties were evaluated using R software. The samples studied gave variable yields, ranging from 0.70 ± 0.03% to 4.12 ± 0.21%. The main constituents of Thymus vulgaris harvested from the municipality of El Hammam are carvacrol (68.8%), γ-terpinene (11.5%), and p-cymene (3.9%), while borneol (41.3% and 31.7%) and carvacrol (14.6% and 9.8%) are the most abundant in Thymus satureioides of the communes of Tata and Tigrigra respectively. For Thymus zygis, the results revealed the dominance of carvacrol (51.7% and 57.5%) for the municipalities of Tigrigra and Ain Aghbal, thymol (47.1% and 42.1%) for the municipalities of Bensmim and Timahdite respectively. These chemical profiles have similarities, but also reveal differences from the results given in the literature. In addition, the essential oils most active towards the microorganisms evaluated were those of Thymus vulgaris, followed by Thymus zygis and Thymus satureioides. These EO have very powerful MIC (MIC ⩽ 300 μg/ml) against Gram-negative bacteria, and in particular, concerning Enterobacters cloacae, Citrobacter koseri, and Acinetobacter baumannii. Thymus zygis EO is the most active on candidates compared to Thymus vulgaris and Thymus satureioides EO, except Candida dubliniensis which was inhibited by Thymus satureioides EO from the commune of Azrou with a MIC = 18.75 μg/ml. The correlation determined between the major components and MIC showed that phenols have the strongest positive effects on antimicrobial properties, followed by terpenes and non-aromatic alcohols. In addition, different sensitivities of pathogens to chemical families have been observed against Enterobacter cloacae, Citrobacter koseri, Candida parapsilosis, Staphylococcus aureus multiresistant, Pseudomonas aeruginosa, Acinetobacter baumannii, and Aspergillus niger. Our results support the idea that these oils could be very useful in flavoring, food preservation, as well as a source of antimicrobial agents of great power against multidrug-resistant strains.

## 1 Introduction

Nowadays, scientific research is oriented towards the development of new applications and the exploitation of the pharmacological properties of essential oils in the fields of pharmacy, cosmetics, and agri-food. Their production and antimicrobial potential have taken an important place in scientific research (Belaqziz et al. 2010).

Due to its geographical location and the diversity of its ecological conditions, Morocco offers a rich and diverse floristic biodiversity and vegetation. A large number of aromatic and medicinal plants grow spontaneously and are used in the traditional pharmacopeia or exploited for industrial needs (Bellakhdar 1997). Among the aromatic and medicinal species that grow spontaneously in Morocco and that are the most exploited and demanded on the international market, are the thyme species, notably *Thymus zygis*, *Thymus satureioides*, and *Thymus vulgaris*.

The name “thyme” includes a group of spontaneous species belonging to the family Lamiaceae and the genus *Thymus*. This genus *Thymus* includes between 250, and 350 species, subspecies, and varieties of wild plants (Lawrence and Tucker, 2002, Napoli et al., 2010). It represents a polymorphic taxon, both chemical and morphological (Morales 2002). According to Jalas (Jalas 1971). *Thymus* is divided into eight sections: *Micantes, mastichina, Piperella, Teucrioides, Pseudothymbra, Thymus, serpyllum,* and *Hypodermis.* Thyme species are distributed between Europe, West Asia, and the Mediterranean. They are widespread in Northwest Africa (Morocco, Tunisia, Algeria, and Libya), they also grow on the mountains of Ethiopia and southwest Saudi Arabia through the Sinai Peninsula in Egypt. They can also be found in Siberia and even the Himalayas. According to the study conducted by Nickavar (Nickavar, Mojab, et Dolat-Abadi 2005), about 110 different species of the genus *Thymus* are concentrated in the Mediterranean basin. Indeed, the Mediterranean region is considered to be the center of this genus. In traditional medicine, the leaves and floral parts of *Thymus* species are widely used as a tonic, antiseptic, antitussive, expectorant, and carminative tea, as well as for the treatment of colds (Ghasemi Pirbalouti et al., 2007, Rasooli and Mirmostafa, 2002). It has been reported that thyme has many biological activities: antispasmodic, antimicrobial, antioxidant, antiplatelet, analgesic, and anti-inflammatory (El Bouzidi et al., 2013, Jamali et al., 2012, Okazaki et al., 2002, Meister et al., 1999, Grosso et al., 2010, Mokhtari et al., 2021).

In Morocco, the genus *Thymus* commonly called “zaîtra” or “Azoukeni” by the local populations is considered a panacea. This genus is represented by 21 species, 10 of which are endemic, this translates into an endemism rate of 47.62% (Fennane et Ibn-Tattou 2007). Some of these species have already been extensively studied while others remain little known. This is particularly the case of *Thymus zygis, Thymus vulgaris,* and *Thymus satureioides.* These species are located mainly in the regions of the High Atlas, Anti-Atlas, Middle Atlas and Middle Atlantic in Morocco (Fennane et Ibn-Tattou 2007). They are generally used against respiratory infections, acute bronchial affections, colds, and as a food preservative (Li et al. 2019). They are also harvested by local populations to be sold as fresh and dried herbs in city markets. However, these species are not widely exploited, export quantities are very limited, and Moroccan thymes remain under severe threat from grazing, overexploitation, or habitat loss. Despite all these obstacles, Morocco is considered the second country where France sources thyme according to the latest report on the Perfume, Aromatic and Medicinal Plants Market 'Panorama 2018′ (FranceAgriMer 2020).

The chemical profile of several varieties of thyme from different regions of Morocco is highly variable, the content and nature of their major compounds vary considerably from one sample to another depending on the origin of the plants. These variations in chemical composition are due to geographical position, harvest season, environmental and climatic conditions, and also genetic factors (Benjilali et al., 1987, El Ajjouri et al., 2008).

Based on the food reports of the U.S. Food and Drug Administration (FDA), the EO of thymes are generally recognized as healthy. Among them, thymol is commonly considered a natural, non-toxic, and ecological bioactive agent, with various pharmacological functions (Lange 2002).

According to the WHO, by 2050, microbial infections could be one of the world's leading causes of death (Dadgostar 2019). Indeed, given the scarcity of new antibiotics, multidrug-resistant microorganisms are jeopardizing WHO's efforts to combat multidrug-resistant infections. In this case, to find effective and accessible alternatives from natural products that are nowadays experiencing a revival of interest and enjoy growing popularity, we have set as the main objective, the highlighting of the potentialities of endemic species of thymes from the Middle Atlas and Anti-Atlas regions of Morocco.

We have in fact evidenced the effect of the origin of seven samples on the yield, chemical composition and antimicrobial activity of essential oils of *Thymus vulgaris*, *Thymus satureioides* and *Thymus zygis* collected from the regions of Khenifra, Tata and Ifrane respectively. We have also determined the chemical composition and evaluated the antimicrobial activity of EOs against twenty-four of the most pathogenic bacteria and eight fungi, which have been shown to be implicated in healthcare-associated and food-borne infections.

This study is integrated in a context of valorization of Moroccan aromatic and medicinal plants that are potentially exploitable.

## 2 Materials and methods

### 2.1 Plant material

**Fig. 1:**
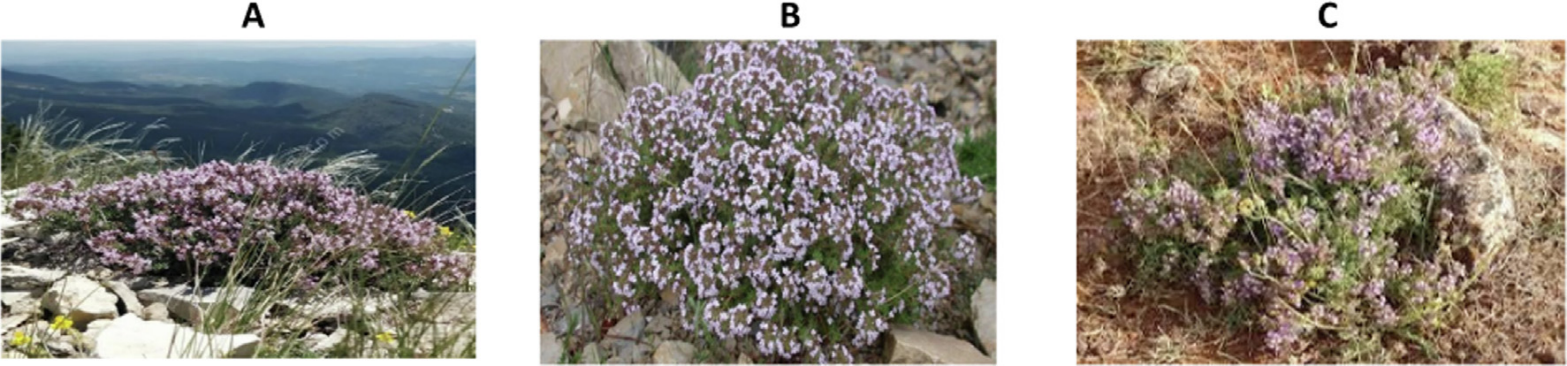
Morphological aspect of the studied thyme species, **A:***Thymus vulgaris*; ***B:****Thymus satureioides* and **C:***Thymus zygis.*

The present study is carried out on samples of three species of thyme from seven sites. These samples were collected at the time of their flowering, at the level of the localities of the regions of Tata, Khenifra, and Ifrane. Then, the plants were dried in the shade for about ten days. They were identified and authenticated at the Scientific Institute of Rabat, Department of Botany. Detailed information on each species is given in the Table 1, Table 2 and Fig. 1.

**Table 1:** Distribution of individuals in the populations of the thyme species studied and their harvest sites by region.

N°	*Latin name*	*Abbreviation*	Harvest site		Parts used	Latitude (x)	Longitude (y)	Altitude (m)	Harvest year
			**Region**	**Locality**					
**1**	*Thymus vulgaris*	*TVHA*	Khenifra	El hammam	Flowering tops	5° 28′ 09″ W	33° 10′ 28″ N	1125	2018
**2**	*Thymus satureioides*	TSAZ	Ifrane	Azrou		5° 12′ 30″W	33° 26′ 25″ N	1499	2018
**3**		*TSTA*	System	System		8° 39′ 56″ W	29° 37′ 04″ N	1625.5	2018
**4**	*Thymus zygis* subsp*. Gracilis*	*TZTIM*	Ifrane	Timahdite		5° 02′ 21″ W	33° 14′ 21″ N	1961.5	2019
**5**		*TZAA*	Ifrane	Ain Aghbal		5° 15′ 33″ W	33° 26′ 30″ N	1170	2018
**6**		*TZTIG*	Ifrane	Tigrigra		5° 18′ 43″ W	33° 24′ 33″ N	1121	2019
**7**		*TZBE*	Ifrane	Bensmim		5° 10′ 33″ W	33° 30′ 29″ N	1579	2019

**Table 2:** The taxonomic classification of the genus *Thymus.*

**Reign**	**Plants**
Kingdom	Plantae
Division	Magnoliophyta
Class	Magnoliopsida
Order	Lamiales
Family	*Lamiaceae*
Genus	*Thymus*

### 2.2 Microbial material

The determination of the antimicrobial activity of different EO was carried out on twenty-four bacterial strains and eight fungal strains (Table 3, Table 4). These selected microorganisms are pathogenic, known for their high resistance, invasive and toxic power in humans. They are frequently encountered in many infections in Morocco that pose a clinical and therapeutic problem. These strains were isolated from the hospital environment: Mohamed V-Meknes Provincial Hospital. While, the antifungal activity was performed on isolates from the Mycothèque of the laboratory of parasitology-mycology of the Hospital Center Ibn Sina - CHIS Rabat. All strains were collected on a 20% glycerol stock at −80 °C, rejuvenated on Mueller Hinton and Sabouraud broths, and subcultured before use.

**Table 3:** List of Bacterial Strains Tested with their references.

**References**	**Gram-positive Cocci**	**References**	**Gram-negative Bacilli**
5994	*Staphyloccocus epidermidis*	7DT2404	*Acinetobacter baumannii*
4IH2510	*Staph aureus BLACT*	3DT1938	*Escherichia coli (sauvage)*
2DT2220	*Staph aureus STAIML/ MRS/ mecA/ HLMUP/ BLACT.*	2DT2057	*Escherichia coli BLSE*
7DT2108	*Streptococcus acidominimus*	07CQ164	*Enterobacter aerogenes*
3EU9286	*Streptococcus groupe D (non-enterococcus)*	02EV317	*Enterobacter cloacae*
7DT1887	*Streptococcus agalactiae*	3DT2151	*Citrobacter koseri*
2EU9285	*Streptococcus porcinus*	3DT1823	*Klebsiella pneumonie ssp pneumonie*
2CQ9355	*Enterococcus faecalis*	2DS5461	*Proteus mirabilis*
13EU7181	*Enterococcuss faecium*	2DT2138	*Pseudomonas aerogenosa*
		5442	*Pseudomonas fluorescence*
		2DT2140	*Pseudomonas putida*
		375BR6	*Serratia marcescens*
		2CG5132	*Salmonella* sp.
		7DS1513	*Shigella* sp.
		ATCC27729	*Yersinia enterocolitica*

**Table 4:** List of fungal Strains Tested with their references.

**GENUS**	**References**	**SPECIES**
*Yeasts*	Ca	*Candida albicans*
	Cky	*Candida kyfer*
	Ckr	*Candida krusei*
	Cpa	*Candida parapsilosis*
	Ct	*Candida tropicalis*
	Cd	*Candida dubliniensis*
	Sacc	*Saccharomyces cerevisiae*
*Molds*	AspN	*Aspergillus niger*

### 2.3 Phytochemical study

#### 2.3.1 Quality control of plant material

##### 2.3.1.1 The moisture content of dry matter

The method used is adopted according to the AFNOR standard (NF - V03-402 1985) (AFNOR 1985). A quantity of 5 g of sample is weighed, in crucibles previously dried in a desiccator and weighed. Solid crucibles are placed in an oven at 103 °C for 24 h. At the end of this time, they are cooled in a desiccator and weighed. The result is expressed as a percentage of dry matter:

$$\mathrm{MC}\%=\left(\frac{M_{0}-M_{1}}{M_{0}}\right)\times 100$$

With: M_0:_ initial mass of the plant; M_1:_ mass after drying.

##### 2.3.1.2 Determination of pH

The principle consists in adding 10 ml of hot distilled water to a quantity of 2 g of the tested sample. The mixture is filtered and left to cool. pH was measured using Ohaus Starter 3100 pH Bench meter equipped with STPURE electrode.

##### 2.3.1.3 Ash content

The principle is based on the calcination of the sample at a temperature of 550° C in a muffle furnace until whitish ash of constant weight is obtained (NFV05-113, 1972)(AFNOR 1972). The organic matter content is calculated by the following formula:

$$\mathrm{OM}\%=\left(\frac{W_{1}-W_{2}}{\mathrm{TS}}\right)\times 100$$

OM%: Organic matter; W_1:_ Weight of the capsule and sample before calcination; W_2:_ Weight of the capsule and the sample after calcination; TS: Test sample.

The ash content is calculated as follows: Ash % = 100 – OM%.

##### 2.3.1.4 Determination of heavy metal: inductively coupled plasma atomic emission spectroscopy (ICP-AES)

The heavy metals involved are arsenic (As), cadmium (Cd), chromium (Cr), iron (Fe), lead (Pb), antimony (Sb), and titanium (Ti). There are limited standards for contamination for each metal. However, exceptions are made for drugs whose raw materials are known to accumulate cadmium in large quantities. For the determination of the contents of major elements (As, Cd, Cr, Fe, Pb, Sb, and Ti), we chose the standardized mineralization protocol (AFNOR, 1999), with aqua regia (HNO_3_ + 3 HCl). The latter allows for large sample sizes, which limits the problems of sample representativeness. The process consists of mixing the crushed plant material (0.1 g) with 3 ml of the aqua regal prepared from 1 ml of nitric acid HNO_3_ (99%) and 2 ml of hydrochloric acid HCl (37%), the whole is placed in a reflux assembly at 200° C for two hours, after cooling we let it settle. Then we took the supernatant and filtered it on a membrane of 0.45 μm and then we completed it to 15 ml with distilled water. The concentrations of heavy metals were determined by the ICP-AES inductively coupled plasma atomic emission spectrometer (Ultima 2 Jobin Yvon) at the UATRS laboratory (Technical Support Unit for Scientific Research) at CNRST-Rabat (Skujins 1998).

#### 2.3.2 Extraction of essential oils from thymes and determination of yields

The essential oils were extracted from the flowering tops of thymes by hydrodistillation using a Clevenger-type apparatus. Indeed, three distillations were made by boiling 100 g of the plant material, of each plant, impregnated with water for three hours. Then the resulting oil was dried by adding anhydrous sodium sulfate (Na_2_SO_4)_ and stored at a temperature of-4 °C in a dark bottle until use. The EO yield was calculated from 100 g of the plant matter by the formula (Akrout et al. 2001):

$$\mathrm{Rdt}\left(\%\right)=\frac{W(EO)}{W0}\times 100$$

With.

W(EO): Weight of EO recovered (g).

W_0_: weight of plant material (100 g).

#### 2.3.3 Analysis and identification of the chemical composition of EO

The chromatographic analysis was carried out on a HP 6890 series gas chromatograph (Hewlett Packard, Palo Alto, CA, USA), equipped with a DB-5 (5% phenylmethylsiloxane) capillary column (30 m × 0.25 mm × 0.25 µm film thickness), an FID detector set at 250 °C and lied by a mixture of H_2_/air gas. The fragmentation is carried out by electron impact of 70 eV intensity. The column temperature is programmed at a rate of 4 °C/min rise from 50 to 200 °C for 5 min. The injection mode is split (leak ratio: 1/70, flow rate ml/min), the carrier gas used is nitrogen with a flow rate of 1 ml/min. The device was controlled by a HP Chemstation computer system managing the operation of the device and allowing to follow the evolution of chromatographic analyses. The GC–MS was coupled to a mass spectrometer (HP 5973 series).

The identification of the chemical composition of the EO was performed by determining and comparing the Kovats Indices (KI) of the compounds with those of known standard products described in the databases of Kovats, E.S. (1965) (Kovats 1965), Adams (Adams 2007) and Hübschmann (Hübschmann 2015).

Identification of each compound was performed using the Kovats index by comparing peak retention times with those of known authentic standards available in the authors' laboratory and comparing their reported KI and MS data with those recorded in the 14 WILEY and NIST standards mass spectra database and published literature. Kovats's indices compare the retention time of any product with the retention time of a linear alkane containing the same carbon number. They are determined by co-injecting a mixture of the alkanes (C7-C40 standard) under the same operating conditions.

### 2.4 Determination of minimum inhibitory concentration, minimum bactericidal concentration, and minimum fungicidal concentration

Determination of the minimum inhibitory concentration (MIC) was performed following the reference method of microdilution using 96-well microplates (Balouiri, Sadiki, et Ibnsouda 2016). The MIC is the lowest concentration of EO that produces complete inhibition of growth appreciable to the naked eye of the bacteria and fungi tested after incubation. Therefore, from a stock solution of the essential oil prepared in 10% DMSO, a series of dilutions were performed to obtain concentrations of 5 to 0.93 10-^2^ mg/ml of each EO. These dilutions were prepared in Mueller-Hinton broth medium for bacteria and in Sabouraud broth for fungi for a final volume of 100 µl for each concentration. Then, 100 µl of microbial inoculum with a final concentration of 10^6^ or 10^4^ CFU/ml in the case of bacteria or fungi were added to the different concentrations of the dilution series. After incubation for 24 h at 37 °C, 10 µl of resazurin is added to each well as an indicator of bacterial growth. After a second incubation at 37 °C for two hours, microbial growth was revealed by the change in coloration from purple to pink. The MIC value is determined as the lowest concentration that prevents a color change of the resazurin. The 11th and 12th wells were considered the growth and sterility control respectively. The test was repeated twice for each oil. The standard antifungal studied was terbinafin 250 mg, this drug was added, after grinding, to 2 ml of 10% DMSO. To determine the minimum bactericidal concentration/minimum fungicidal concentration (MBC/MFC), 10 µl was taken from each well with no visible growth and plated on Mueller Hinton (MH) agar for 24 h at 37 °C for bacteria or on Sabouraud for fungi. BMC and MHC were defined as the lowest concentration of samples tested that produced a 99.99% reduction in CFU/ml compared to the control. In addition, the MBC/MIC or MFC/MIC ratio of each extract can be calculated to assess the antimicrobial potency, thus, if the ratio is<4, the effect of the essential oil is bactericidal/fungicidal, if the ratio is>4, the sample has a bacteriostatic/fungistatic effect (Bissel et al. 2014).

### 2.5 Statistical analysis

All data are presented as the average (S.E.M) of the indicated number of experiments. The results were analyzed by Unidirectional or Bidirectional Variance Analysis (ANOVA) with a Tukey-Kramer multiple comparison post-test performed using Graph Pad Prism version 8.0 for Windows (Graphpad Software Inc. San Diego. California, USA). The statistical significance was set at p < 0.05. The results relating to the chemical variability of EO were analyzed using the R software. The Principal Component Analysis (PCA) was carried out with Pearson type matrices. The Hierarchical Cluster Analysis (HCA) and the dendrograms were made with dissimilarity matrices calculated in Euclidean distance and the method of aggregation systematically chosen is the average link. To assess whether the constituents of essential oils can be useful in reflecting the relationships between phytochemical and antimicrobial activities, 20 major compounds detected in the oil samples were selected and used for this purpose. The major compounds and all MIC values of essential oils were subjected to PCA and HCA.

## 3 Results and discussion

### 3.1 Phytochemical study

#### 3.1.1 Quality control of plant material

The collected samples have undergone quality control at our laboratory level by measuring several characteristic parameters such as moisture content (MC), pH, ash, and ICP (Analysis of elemental impurities). The results are presented in Table 5, Table 6.

**Table 5:** Quality control of plant matter (MC, pH and Ash).

***Species***	***MC (%)***	***pH***	***Ash (%)***
**TSAZ**	14.48	5.17	7.62
**TSTA**	14.27	5.69	7.46
**TVHA**	18.88	5.46	8.40
**TZAA**	17.43	5.21	6.39
**TZTIM**	14.80	5.26	7.34
**TZTIG**	14.86	5.28	6.78
**TZBE**	15.00	5.30	14.31

**Table 6:** Concentration of heavy metals (mg/l) (ICP) and FAO/WHO Maximum Limit (2009).

**Species**	Arsenic (As)	Cadmium (Cd)	Chrome (Cr)	Iron (Fe)	Lead (Pb)	Antimony (Sb)	Titanium (Ti)
*TVHA*	0,1272	0,0442	0,0691	0,6034	0,1059	0,1225	0,0742
*TZAA*	0,004	0,001	0,0200	4,489	0,0878	0,0050	0,0300
*TZTIM*	0,1288	0,0438	0,0657	0,7219	0,0998	0,1195	0,0722
*TZTIG*	0.0025	0.0016	< =0.001	< =0.001	< =0.001	< =0.001	< =0.001
Maximum limit	1	0,3	2	20	3	1	–

##### 3.1.1.1 Moisture content

The moisture content of thymes measured by the oven method varies between 14.27 and 18.88%. These moisture contents are directly related to the activity of the water (Aw). The high contents are recorded for *Thymus vulgaris* (18.88%) collected from El Hammam locality in the Khenifra region, followed by the species of *Thymus zygis* (17.43; 15; 14.86 and 14.80%) collected from the Ifrane region in Ain Aghbal, Bensmim, Tigrigra and Timahdite localities respectively. While the moisture content recorded for *Thymus satureioides* (14.48 and 14.27%) collected from Tata and Tigrigra localities respectively are the least low.

In addition, most microorganisms grow at a water activity between 0.95 and 1, molds and most bacteria are unable to grow at a moisture content below 24% where Aw is equal to 0.7. Therefore, samples dried under laboratory conditions are of good quality.

##### 3.1.1.2 pH

To know the stability of the plants concerning the microorganisms and the assimilability of the mineral elements, the pH of the plant matter was determined, it is a quality index determining the aptitude for the conservation of the plants. It is one of the main obstacles that microbial flora must overcome to ensure its proliferation. Microorganisms pathogenic to humans rarely grow at a pH acid, below 4. Most microorganisms develop at pH levels close to neutrality. Moreover, the pH conditions the assimilability and the good availability of mineral elements. Acidophilic plants, or so-called heathland plants, require a pH < 5.5 and are free of total limestone, whereas neutrophilic plants can tolerate a wide range of pH (by convention between 6,0 and 6,5) (Hamani et al., 2006, Oteng-Gyang, 1984).

Including our case, the pH of the species studied varies between 5.17 and 5.69. These values offer these thymes a quality of good character with good availability of mineral elements.

##### 3.1.1.3 Ash content

The percentage of total ash provides information on the mineral content, as minerals are not transformed into volatile substances at high temperatures, unlike organic matter. The total ash contents observed are comparable and similar between the species studied except for *Thymus zygis* collected in the Bensmim region of Ifrane in which we recorded a high rate of about 14.31%, the other rates vary between 6.39 and 8.40%. The results of our samples are in the limits, since the international food standards “Codex Alimentarius C.X.S 328-2017″ for dried thyme are set at 12%.

##### 3.1.1.4 Heavy metal assays

Medicinal herbs are easily contaminated during growth, development, and processing. After collection and processing, the heavy metals contained in plants eventually enter the human body and can disrupt the normal functions of the central nervous system, liver, lungs, heart, kidneys, and brain, leading to hypertension, abdominal pain, rashes, intestinal ulcers and different types of cancers.

The Table 6 shows the average concentration of various metals measured in the powders of the flowering tops of thyme. All samples tested had values below the limit value regulated by FAO/WHO. Moreover, we note that the concentrations of heavy metals at the level of the different thymes are low except *Thymus zygis* collected in Ain Aghbal (TZAA) locality of the Ifrane region in which we observed a slightly high concentration of Iron (4.489 mg / l) compared to other species. This slightly elevated amount of Iron in TZAA may be due to foliar absorption of ambient air. These results show a slight variation in the concentrations of heavy metals in the powders of the species evaluated. These variations depend on the species and the assimilated element. The accumulation capacity of metals Cr, Pb, Sb, Ti by the studied thymes follows the following order: TVHA > TZTIM > TZAA > TZTIG. However, the order of accumulation of heavy metals As, Cd and Fe is variable from one species to another.

By way of conclusion, the thymes studied can be proposed for direct consumption, as an ingredient in food processing, or for repackaging if necessary, as they can be safely intended for industrial treatments.

#### 3.1.2 Yield and density of essential oils

The average essential oil yield of each species was calculated according to the dry plant matter obtained from the flowering tops of the different thymes. The essential oil yield obtained is shown in Table 7.

**Table 7:** Yields (ml/100 g dry matter) and density of essential oils of the seven thyme samples.

**Species**	**Yield (%)**	**Density (g/ml)**
*TVHA*	3,77 ± 0,19	0,953
TSAZ	1,4 ± 0,07	1,113
*TSTA*	0,7 ± 0,03	1,033
*TZTIM*	2,68 ± 0,13	0,863
*TZAA*	3,27 ± 0,16	0,943
*TZTIG*	3,32 ± 0,17	1,013
*TZBE*	4,12 ± 0,21	0,883

The density of each essential oil analyzed by the conventional method ranged from 0.863 to 1.113 g/mL, as shown in Table 7.

According to this study, it is found that *Thymus zygis* (TZBE) collected in Besmim, Ifrane region at an altitude of 1579 m, gave the highest essential oil yield (4.12%) followed by the *Thymus vulgaris* (TVHA) sample collected at an altitude of 1125 m in El Hammam, Khénifra region which is (3.77%). A high EO yield (3.32; 3.27 and 2.68%) was also obtained for *Thymus zygis* species (TZTIG, TZAA, and TZTIM) collected in the Ifrane region at Tigrigra (1121 m), Ain Aghbal (1170 m), and Timahdite (1961.5) respectively. For *Thymus satureioides* (TSAZ and TSTA), the average essential oil yield is (1.4%) for Ain Aghbal at an altitude of 1499 m and (0.7%) at Tata located at an altitude of 1625.5 m. These two rates are low compared to those obtained for Thymus *zygis* and *Thymus vulgaris* samples. On the other hand, the yields obtained for the different thymes are relatively important compared to some plants that are exploited industrially as a source of essential oils.

The values of the EO yields of *Thymus satureioides* from Ifrane (Morocco), reported by Elouali Lalami, A. and co-workers (LALAMI et al. 2013) is close to 1.1%, this value is in agreement with that found in our study on *Thymus satureioides* from Tata (Morocco). Salaheddine El-Bakkal and co-workers (El-Bakkal et al. 2020) found a higher yield of around 2.39% on *Thymus satureioides* from Marrakech (Morocco).

For *Thymus vulgaris,* our results are significantly higher than those obtained by Elouali Lalami (LALAMI et al. 2013) and by B, Imelouane, and co-workers (Imelouane et al. 2009) who recorded a yield of around 0.5 and 1% respectively in the regions of Ifrane and Berkane (Morocco).

In addition, the EO yields of *Thymus zygis* are comparable to the yields recorded at three different sites in Morocco (El Hajeb, Azrou, and Timahdite) by Souad Yakoubi and co-workers (Yakoubi, Cherrat, Diouri, Hilali, et al. 2014), but remain slightly lower than that recorded in Azrou by Radi Fatime Zahra and co-workers (5.25%) (Radi et al. 2022).

From these results, we can deduce that intra- and interspecific variations in yields appear to correlate with abiotic factors such as specific climate in the sample collection regions and geographical factors such as altitude and soil nature. This is consistent with the results reported by El Idrissi and co-workers (El idrissi et al. 2016), according to which, variations in EO yields can be due to several factors, including climate, altitude, and the nature of the soil. Several studies have shown the influence also of the vegetative cycle, the nature of the plant (dried or fresh), the picking period as well as the mode of extraction on the yield and quality of the essential oil (Drioiche et al., 2020, Jordán et al., 2006).

#### 3.1.3 Chemical composition of thyme essential oils

**Fig. 2:**
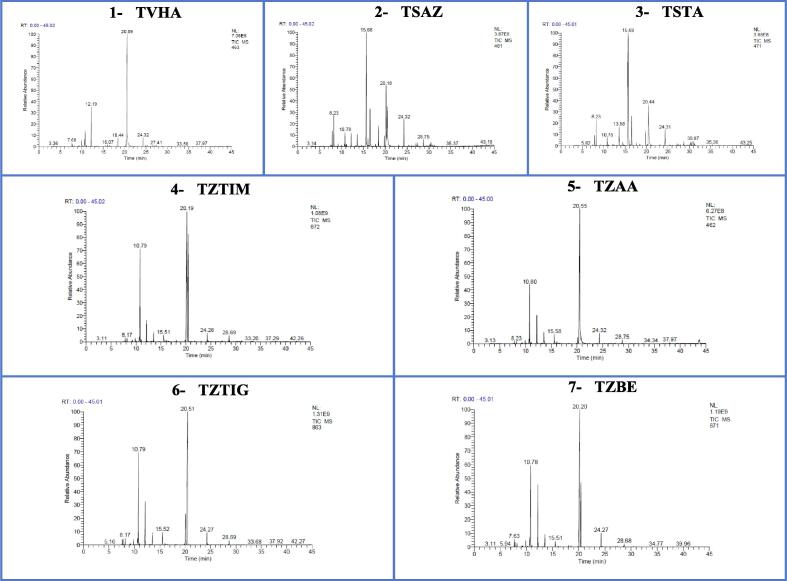
Chromatographic profiles of the studied essential oils, **(1-** TVHA: *Thymus vulgaris from* El Hammam; **2 & 3-** TSAZ and TSTA: *Thymus satureioides* from Azrou and Tata; **4, 5, 6 & 7-** TZTIM, TZAA, TZTIG and TZBE: *Thymus zygis* of Timahdite, Ain Aghbal, Tigrigra and Bensmim).

The present study revealed the presence of a high level of variation in the chemical composition of the EO of thyme species collected in the Middle Atlas region (Khenifra, Ifrane) and the Anti-Atlas (Tata region). The GC/MS analyses of the EO of the studied thymes have allowed to make the chromatographic profiles illustrated in Fig. 2, and to identify the different chemical compounds listed in Table 8.

**Table 8:** Chemical profiles of the essential oils of the studied thymes.

**KI**	**Compounds**	**TVHA**	**TSAZ**	**TSTA**	**TZTIG**	**TZBE**	**TZAA**	**TZTIM**
926	Tricyclene	–	0,2	0,2	–	–	–	–
**930**	**α-Thujene**	**0,8**	**0,2**	**–**	**0,6**	**0,9**	**–**	**0,1**
**939**	**α-Pinene**	**0,5**	**2,4**	**2,1**	**–**	**0,5**	**0,5**	**0,4**
**954**	**Camphene**	**0,1**	**4,9**	**5,6**	**0,8**	**0,4**	**0,5**	**0,4**
979	β-Pinene	0,1	0,4	0,2	0,1	0,1	0,1	0,1
**979**	**1-Octen-3-ol**	**0,3**	**–**	**–**	**0,2**	**0,1**	**0,2**	**0,1**
**990**	**Myrcene**	**1,4**	**0,1**	**–**	**0,7**	**0,8**	**0,5**	**0,3**
1002	α-Phellandrene	0,2	–	–	0,1	0,1	0,1	0,1
1011	δ-3-Carene	–	–	–	0,1	0,1	–	–
**1017**	**α-Terpinene**	**1,8**	**0,4**	**0,1**	**0,7**	**1,6**	**1,1**	**0,5**
**1024**	**p-Cymene**	**3,9**	**2,4**	**1,8**	**19,0**	**15,9**	**14,8**	**18,4**
1029	Limonene	0,2	0,3	0,1	0,2	0,2	0,2	0,2
1029	β-Phellandrene	0,2	–	–	0,1	0,1	0,1	–
1031	1,8-Cineole	–	0,1	–	–	–	–	0,2
1050	(E)-β-Ocimene	0,2	–	–	–	–	–	–
**1059**	**γ-Terpinene**	**11,5**	**2,3**	**0,3**	**7,5**	**11,9**	**7,2**	**3,1**
1070	*cis*-Sabinene hydrate	0,1	–	–	0,4	0,4	–	0,1
1072	*cis*-Linalool oxide	–	–	0,2	–	–	0,1	–
1085	*meta*-Cymenene	–	–	–	–	–	–	0,1
1086	*trans*-Linalool oxide	–	–	–	0,1	–	–	–
1088	Terpinolene	0,1	0,1	–	0,1	0,1	–	0,1
**1096**	**Linalool**	**0,1**	**2,3**	**4,9**	**1,8**	**1,8**	**3,1**	**1,3**
1098	*trans*-Sabinene hydrate	–	–	–	0,1	–	–	–
1139	*trans*-Pinocarveol	–	–	0,2	–	–	–	–
1146	Camphor	–	0,2	0,7	–	–	–	–
**1169**	**Borneol**	**0,3**	**31,7**	**41,3**	**2,3**	**0,9**	**2,5**	**1,1**
**1177**	**Terpinen-4-ol**	**0,5**	**1,2**	**1,1**	**0,2**	**0,1**	**0,5**	**0,2**
**1188**	**α-Terpineol**	**0,4**	**7,9**	**8,2**	**0,1**	**0,1**	**–**	**0,1**
1233	Pulegone	–	–	–	–	–	–	0,3
1235	Thymol, methyl ether	–	–	–	0,1	0,2	–	–
1239	Isobornyl formate	–	0,3	0,7	–	–	–	–
**1244**	**Carvacrol, methyl ether**	**2,5**	**3,8**	**0,4**	**0,1**	**0,1**	**–**	**–**
1270	(E)-Cinnamaldehyde	–	–	–	–	0,2	–	–
**1285**	**Bornyl acetate**	**–**	**2,1**	**3,6**	–	–	–	–
**1290**	**Thymol**	**0,5**	**14,8**	**0,3**	**8,4**	**47,1**	**1,8**	**42,1**
**1299**	**Carvacrol**	**68,8**	**9,8**	**14,6**	**51,7**	**12,0**	**57,5**	**26,9**
1343	Piperitenone	–	–	–	–	–	–	0,1
1368	Piperitenone oxide	–	–	–	–	–	–	0,1
1372	Carvacrol acetate	0,1	–	–	–	0,1	0,1	–
1376	α-Copaene	–	0,2	0,2	–	–	–	–
**1419**	**(E)-Caryophyllene**	**2,6**	**5,3**	**3,9**	**1,9**	**2,2**	**2,7**	**1,3**
1441	Aromadendrene	0,4	–	–	–	–	–	–
1454	α-Humulene	0,2	0,2	–	0,1	0,1	0,1	–
1466	9-epi-(E)-Caryophyllene	–	–	0,2	–	–	–	–
1479	γ-Muurolene	–	0,1	–	–	–	–	–
1496	Viridiflorene	0,3	–	–	–	–	–	–
1513	γ-Cadinene	0,3	0,5	0,6	0,1	0,1	0,1	0,1
1523	δ-Cadinene	0,3	0,6	0,4	0,1	0,1	0,1	0,1
1555	Thymohydro quinone	–	–	–	0,2	0,1	–	0,1
1578	Spathulenol	0,1	–	0,4	0,1	0,1	–	0,1
**1583**	**Caryophyllene oxide**	**–**	**1,5**	**1,1**	**0,7**	**0,5**	**1,2**	**1,0**
1607	β-Oplopenone	–	–	0,2	–	–	–	–
1640	Caryophylla-4(12),8(13)-dien-5α-ol	–	0,2	0,6	–	–	–	–
**1640**	**Caryophylla-4(12),8(13)-dien-5β-ol**	**–**	**0,5**	**1,0**	–	–	–	–
1640	epi-α-Cadinol	–	0,8	0,9	–	0,1	–	–
**1641**	**allo-Aromadendrene epoxide**	**–**	**0,4**	**1,5**	–	–	–	–
1686	Germacra-4(15),5,10(14)-trien-1-α-ol	–	0,5	0,7	–	–	–	0,2

**Hydrocarbon monoterpenes**		21,5	13,8	10,6	30,3	33,1	25,1	23,4
**Oxygenated monoterpenes**		73,6	74,8	76,6	65,2	62,8	66,1	73,1
**Hydrocarboned sesquiterpene**		3,6	6,0	4,4	2,3	2,3	2,7	1,4
**Oxygenated sesquiterpenes**		0,1	4,1	6,7	0,9	0,9	1,2	1,4
**Total**		**98,8**	**98,7**	**98,3**	**98,7**	**99,1**	**95,1**	**99,3**

By analyzing the main chemical classes of the essential oils studied (Table 8), we found that the thyme samples have EO rich in oxygenated monoterpenes (62.8 to 76.6%) whose majority compound is carvacrol (9.8 to 68.8%) followed by thymol (0.5 to 47.1%), then comes borneol (0.3 to 41.3%) and a low percentage α-terpineol (0.1 to 8.2%). They are also characterized by a high level of hydrocarbon monoterpenes (10.6 to 33.1%) of which the most abundant are represented by p-cymene as the majority compound (1.8 to 19%), γ-terpinene (0.3 to 11.9%), and in small percentage camphene (0.1 to 5.6%). As for hydrocarbon sesquiterpenes, the percentages vary from 1,4 to 6% and they are mainly represented by (E)-caryophyllene (1.3 to 5.3%). Oxygenated sesquiterpenes come last, by rates that vary from 0.1 to 6.7%, with caryophyllene oxide (0.7 to 1.5%) as the majority compound. The latter chemical class is absent for the TVHA sample.

Based on the results presented in Table 8, we note that the EO extracted from *Thymus vulgaris* from Khenifra is represented by the chemotype carvacrol (68.8%), γ-Terpinene (11.5%), and p-Cymène (3.9%).

As for *Thymus satureioides* EO from two sites (Tata and Azrou), they have borneol (41.3% and 31.7%), carvacrol (14.6% and 9.8%) as the majority compounds, followed by α-terpineol (8.2 and 7.9), (E)-caryophyllene (3.9 and 5.3%), camphene (5.6% and 4.9%) and linalool (4.9% and 2.3%). On the other hand, thymol presents 14.86% in the EO of Azrou and only traces in that of Tata.

In *Thymus zygis* EO*,* we found two different chemotypes; carvacrol and thymol. Thymus *zygis EO from* Tigrigra and Ain Aghbal are dominated by carvacrol representing respectively 51.7% and 57.5%. While those of Bensmim and Timahdite, are rich in thymol whose values are respectively 47.1% and 42.1%. We note the presence of other compounds with relatively large proportions such as p-cymene and γ-terpinene.

Compared to chemical compositions published in the literature, the chemical profile described in this work shows similarities but also reveals differences. Indeed, our results confirm those of Fachini-Queiroz (Fachini-Queiroz et al. 2012), Chraibi (Chraibi et al. 2016) and their collaborators who identified carvacrol and borneol as majority compounds for Thymus vulgaris and Thymus satureioides respectively. Furthermore, the results obtained in our work for Thymus zygis are consistent with those of Cherrat et al., 2018 who identified the same majority compounds for the species collected in the Timhdit region (Middle Atlas Morocco). However, the work of Yacoubi et al. performed on Thymus zygis from three regions of the Middle Atlas showed that the three samples are mainly composed of carvacrol (16.1 to 74.3%), thymol (1.5 to 32.5%), p-cymene (6.9 to 40.3%) and γ -terpinene (2.7 to 22%) (Yakoubi, Cherrat, Diouri, EL Hilali, et al. 2014). While the essential oil of Thmus zygis from Meknes is dominated by thymol (44.2%), p-cymene (15.5%) carvacrol (13.5%) and γ-terpinene (11.3%), (Lemrhari et al. 2015). In addition, we found more or less important differences in the chemical composition of the essential oil of Thymus zygis collected in the region of ozoud-azilal (High Atlas-Morocco). It is dominated by thymol (34.1%) and borneol (25.3%) (Jaafari et al. 2007). The essential oil of Thymus zygis in Morocco is therefore characterized by its abundance of carvacrol or thymol (isomeric compounds).

The comparison of the chemical composition of thyme EO shows a diversity of compounds identified both qualitatively and quantitatively. It is impossible to find a chemically homogeneous and standardized EO for *Thymus* species in their natural habitat (Aicha, Rachida, et El Abdelmalek 2013).

**Fig. 3:**
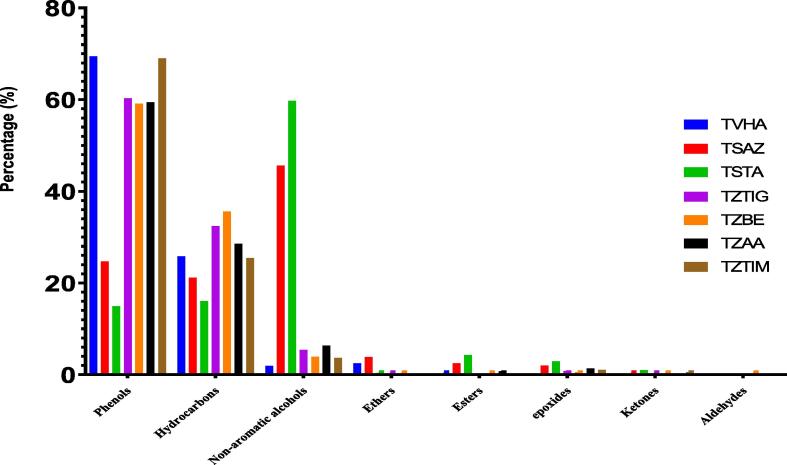
Distribution of chemical families identified in the essential oils of the thymes studied (%).

The number of sesquiterpenes is very low, while the number of monoterpenes is the largest. Also, for all the chemical compounds identified within the essential oils of thyme species, only carvacrol remains the major common component omnipresent in varying quantities. The compounds identified in the different thymes are found in very variable percentages. Moreover, the essential oils of *Thymus vulgaris* and *Thymus zygis* are dominated mainly by phenols followed by hydrocarbons. In contrast, *Thymus satureioides* EO is composed mainly of non-aromatic alcohols, followed by phenols and hydrocarbons. In addition, we find other families of chemical compounds that characterize thyme species such as ethers, esters, aldehydes, epoxides, and ketones with varying proportions (Fig. 3).

The results obtained show valuable variations in the chemical composition of different species, within the same species and between provenances. These differences are remarkable in particular by the nature of the most abundant compound and the other constituents identified. Moreover, even if we find some similar elements, this variability from one locality to another confirms that the chemical profile of the species studied is very variable depending on the origin of the plant, altitude, climate, genetic heritage, and abiotic factors (El El Ajjouri et al., 2008, Ouyahya and Viano, 1984).

### 3.2 Analysis of similarity between species by hierarchical ascending and principal component (PCA) analysis

The analysis of the chemical composition of the essential oils of the studied thymes (*Thymus vulgaris*, *Thymus zygis*, and *Thymus satureioides*); shows a strong diversity of the identified compounds both qualitatively and quantitatively. To obtain a statistical description of our sample and to be able to highlight possible chemical variability and identify possible relationships between the abundance of volatile organic compounds and abiotic and geographical factors, we used two of the most common tests of multivariate descriptive statistics including a hierarchical ascending analysis (HCA) and a principal component analysis (PCA).

#### 3.2.1 Hierarchical cluster analysis (HCA)

**Fig. 4:**
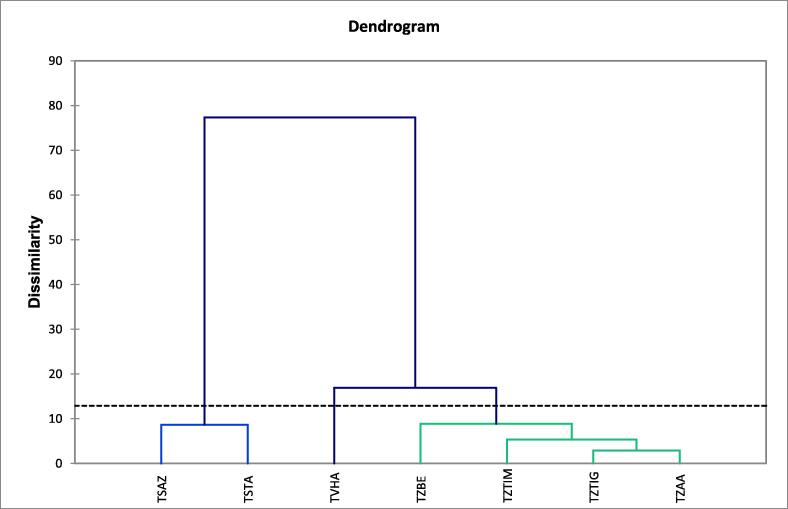
Phylogenetic tree of the studied thyme EO.

First, a hierarchical bottom-up analysis (HCA) was performed to divide the sample into groups of homogeneous observations, each group being well differentiated from the others. This analysis will then combine the samples to produce a dendrogram or classification tree (Fig. 4).

Fig. 4 shows the dissimilarity between the EO samples of the thymes studied. This analysis shows that samples can be grouped into three distinct groups depending on the distance between them. The first group (I) includes the two samples of *Thymus satureioides* (TSAZ and TSTA), the second group (II) contains a sample of *Thymus vulgaris* oil (TVHA). Finally, the third group (III) includes the four samples of *Thymus zygis* (TZAA, TZTIG, TZTIM, and TZBE).

At the level of the dendrogram, we note that the EO of the group (II) includes two samples of EO and are grouped within the same class, so there is an intra-class homogeneity; this is confirmed by the small distance between the two populations, which explains that the two taxa are similar or very close at the level of the chemical profile, for example, species TSAZ presents 91% similarity with species TSTA. On the other hand, groups (III) and (I) are composed of TZAA, TZTIG, TZTIM, TZBE and TVHA. The species of group (III) are heterogeneous concerning class (I), with a significant distance, the species TVHA presents just 17% dissimilarity with the other species (TZAA, TZTIG, TZTIM, and TZBE).

#### 3.2.2 Principal component analysis (PCA) of the majority compounds identified in the essential oils of the thymes analyzed

**Fig. 5:**
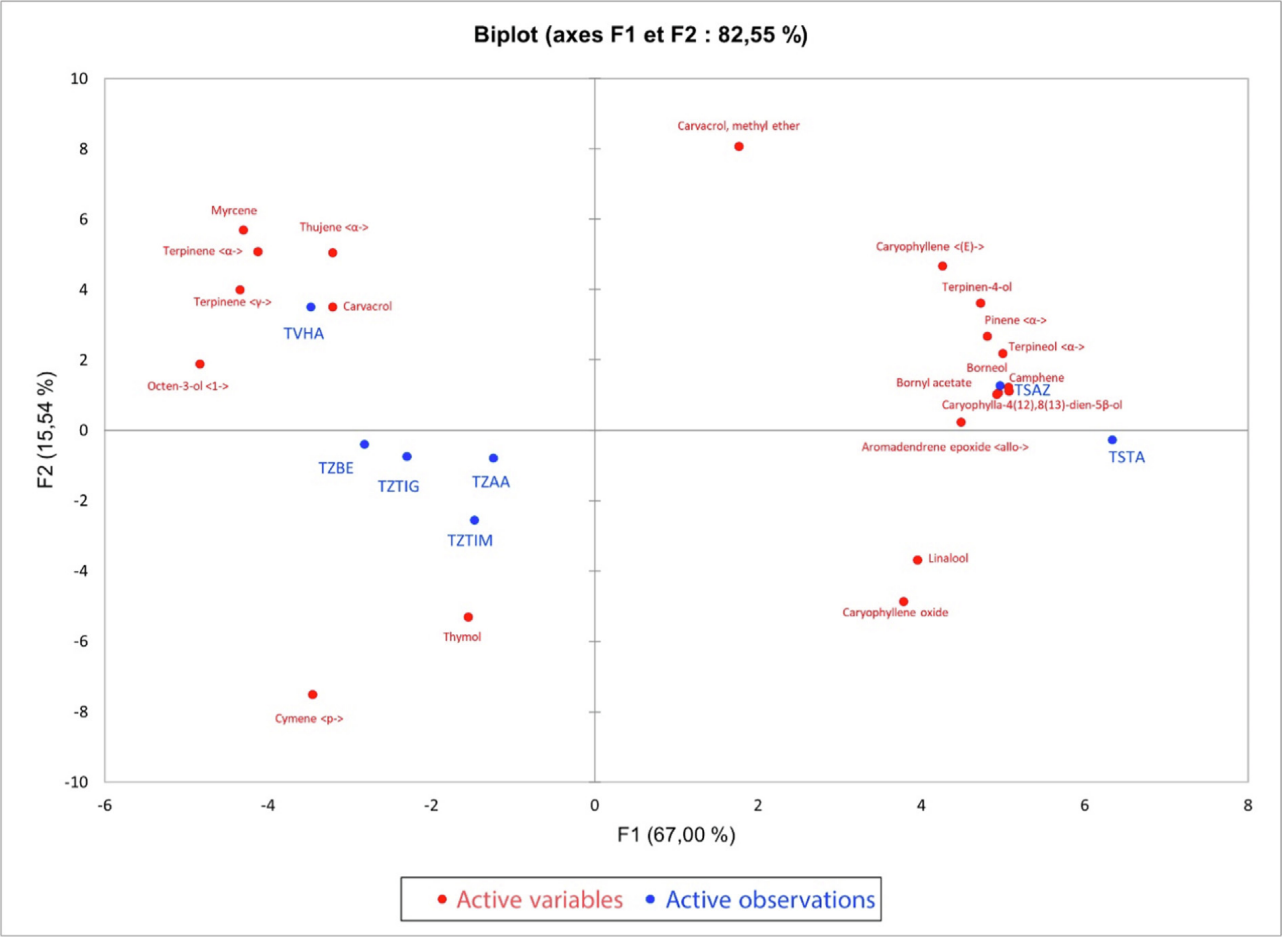
Graphical approach of the principal component analysis according to the plan (F1xF2) of the essential oils of thymes from the different regions studied.

Principal Component Analysis (PCA) is a method that simplifies data by studying the relationships between all variables to determine similarities and dissimilarities between individuals (Fig. 5).

To perform this analysis, we chose the first two factorial axes. The dispersion of thyme species in the plane formed by these two axes concerning the selected variables explains 82.55% of the variability, of which 15.54% on the first axis and 67% on the second axis (Fig. 5). This figure confirms the results of the HCA and highlights the main quantitative differences between the three chemical composition groups (species). Similarly, it shows that the chemical composition of the essential oils of the different populations shows heterogeneity. On the other hand, some chemical compounds are positively correlated, for example, essential oils (TSAZ, TSTA) that are rich in borneol contain less p-cymene and γ-terpinene. Thus, essential oils (TZBE, TZTIG, TZAA, TZTIM) which are rich in Thymol and carvacrol are also rich in p-cymene and γ-terpinene. We note that some species have a similar composition such as species: TZBE, TZTIG, TZAA, TZTIM, and TVHA. On the other hand, others present a significant difference in chemical composition, these are the species TSTA and TSAZ.

We deduce that the chemical composition of the samples of essential oils of thymes collected in different regions of Morocco presents a heterogeneity; indeed, some samples contain carvacrol as the majority compound, while others contain borneol and/or thymol, p-cymene, and γ-terpinene.

Previous studies have found similar chemical compositions, for the essential oils of the three species of thymes in our study namely the presence of carvacrol as a major constituent in the EO of *Thymus vulgaris* of El Hammam of the province of Mrirt which has already been mentioned in some populations of *Thymus vulgaris* in Morocco (El Hattabi et al. 2016)*,* in Brazil (Fachini-Queiroz et al. 2012), in Italy (Vinciguerra et al. 2019). In addition, the dominance of the borneol/carvacrol chemotype for the *Thymus satureioides* of the commune of Tata and the commune of Tigrigra with similar percentages in other work carried out in Morocco, at the level of the regions of Al Haouz; Ljoukak (Ou-Yahia et al. 2017) and Taghbart (Zenasni 2014) as well as that recorded in the Marrakech region (El Bouzidi et al. 2013). Similarly, for *Thymus zygis* subsp. Gracilis CG/MS analyses reveal the dominance of the carvacrol/thymol/p-cymene and γ-terpinene chemotypes. Our results for *Thymus zygis* are similar to other work done in Morocco (Yakoubi, Cherrat, Diouri, Hilali, et al. 2014), Portugal (Miguel et al. 2015), and Algeria (Kouache et al. 2017).

### 3.3 Antimicrobial activity

#### 3.3.1 Antibacterial activity

The results of antimicrobial activity of different Thymes are shown in Table 9. The MIC of the essential oils were classified according to the criteria proposed by Sartoratto, Duarte, Wang, Oliveira, and their collaborators (De Oliveira Pedro et al., 2013, Duarte et al., 2007, Sartoratto et al., 2004, Wang et al., 2017). The antimicrobial activity was classified as high (MIC < 600 μg/ml), moderate (MIC between 600 and 2500 μg/ml), low (MIC > 2500 μg/ml). The CMI and CMB analyses of the different species show a high bactericidal quality of the essential oils against the different strains tested, except *Staphylococcus epidermidis, Streptococcus agalactiae* (B), Streptococcus *porcinus,* Enterococcus *faecium* which showed resistance to the concentrations of EO studied.

**Table 9:** MIC and MBC values (μg/ml) of the essential oils studied.

**Microorganisms**		**TVHA**		**TSAZ**		**TSTA**		**TZTIG**		**TZBE**		**TZAA**		**TZTIM**	
		**MIC**	**MBC**	**MIC**	**MBC**	**MIC**	**MBC**	**MIC**	**MBC**	**MIC**	**MBC**	**MIC**	**MBC**	**MIC**	**MBC**
**Gram-positive Cocci**	***Staphylococcus epidermidis***	1200	2500	2500	2500	2500	2500	2500	2500	2500	2500	1200	1200	1200	5000
	***Staphylococcus aureus BLACT***	300	600	2500	2500	> 5000	> 5000	600	600	1200	1200	600	600	1200	1200
	***Staphylococcus aureus STAIML / MRS / mecA / HLMUP/ BLACT***	300	600	300	600	> 5000	> 5000	> 5000	> 5000	> 5000	> 5000	5000	5000	5000	5000
	***Streptococcus acidominimus***	150	300	300	600	600	600	600	1200	300	600	150	300	300	300
	***Streptococcus group D***	600	600	600	600	2500	2500	1200	2500	600	600	1200	2500	1200	2500
	***Streptococcus agalactiae (B)***	2500	2500	> 5000	> 5000	600	1200	2500	2500	5000	5000	600	1200	2500	2500
	***Streptococcus porcinus***	2500	5000	5000	5000	2500	2500	1200	2500	1200	1200	2500	2500	5000	5000
**Gram-negative Bacilli**	***Escherichia coli sauvage***	300	600	600	1200	75	150	300	600	300	300	600	600	75	150
	***Escherichia coli BLSE***	1200	1200	2500	2500	1200	1200	600	1200	5000	5000	600	600	600	1200
	***Enterobacter aerogenes***	300	300	600	600	2500	5000	300	600	150	300	300	600	150	300
	***Enterobacter cloacae***	18,75	37,5	37,5	75	150	300	18,75	37,5	75	150	75	150	20	37,5
	***Enterococcus faecalis***	300	300	300	300	600	600	300	600	300	600	75	150	600	600
	***Enterococcus faecium***	2500	5000	5000	5000	5000	5000	2500	2500	2500	5000	5000	5000	5000	5000
	***Citrobacter koseri***	18,75	37,5	150	150	75	150	18,75	37,5	150	150	75	150	150	300
	***Klebsiella pneumoniae***	150	300	1200	2500	1200	1200	300	600	600	1200	150	300	300	300
	***Proteus mirabilis***	150	300	1200	1200	2500	5000	300	300	1200	1200	600	1200	600	600
	***Pseudomonas aeruginosa***	150	300	600	1200	37,5	75	300	300	300	300	37,5	75	150	150
	***Pseudomonas fluorescens***	300	300	1200	2500	2500	5000	600	1200	600	600	600	600	600	600
	***Serratia marcescens***	300	600	600	600	1200	2500	150	300	300	600	600	1200	300	300
	***Salmonella sp,***	300	300	1200	1200	600	1200	600	600	600	1200	300	600	300	600
	***Shigella sp***	600	1200	300	600	2500	2500	600	600	600	1200	300	600	600	1200
	***Yersinia enterocolitica***	300	600	1200	1200	5000	5000	600	1200	300	300	300	600	2500	5000
	***Pseudomonas putida***	1200	1200	1200	1200	2500	2500	2500	2500	2500	2500	1200	1200	150	300
	***Acinetobacter baumannii***	75	150	75	150	300	300	150	300	75	150	75	150	37,5	75

As can be noted in this discovery, the essential oils of the thyme species tested showed a remarkable antimicrobial effect towards all the microorganisms studied; in particular *Thymus vulgaris,* EO which showed very high efficacy and inhibited microbial growth from a very low concentration (MIC = 18.75 μg/ml) for certain strains of Enterobacteriaceae, followed by *Thymus zygis* and *Thymus satureioides* species. The EO of the thymes evaluated is very powerful against gram-negative bacteria and in particular, the species *Enterobacter cloacae, Citrobacter koseri, Acinetobacter baumannii.* This potency of EO against Gram-negative germs is sometimes better than that of the antibiotics tested (Table 10). In addition, the essential oil of Thymus *vulgaris* (carvacrol profile) remains the most effective compared to other chemical profiles against the strains tested and in particular against Gram-positive bacteria. Indeed, at a concentration of<300 μg/ml, this chemical profile showed bactericidal activity on all the strains tested, even for the strain Acinetobacter *baumannii* and *Staphylococcus aureus* multidrug-resistant which are species increasingly involved in infections in certain hospital areas such as intensive care units.

**Table 10:** MIC (μg/ml) of antibiotics evaluated by BD Phoenix for selected species.

**Microorganism**		**BD Phoenix™ Instrument Strain References**	**CMI (µg/ml) Identification and antibiogram** instrument BD Phoenix™				
			**Gentamicin**	**Amoxicillin -Clavulanate**	**Vancomycin**	**Trimethoprim-Sulfamethoxazole**	**Penicillin G**
***Gram-positive Cocci***	***Staphylococcus epidermidis***	5994	2		> 8	> 4/76	
	***Staphylococcus aureus BLACT***	4IH2510	< 0.5		2	< 10	
	***Staphylococcus aureus STAIML / MRS / mecA / HLMUP/ BLACT***	2DT2220	2		> 8	> 4/76	
	***Streptococcus acidominimus***	7DT2108	< =250		< 0.5		0,03
	***Streptococcus group D***	3EU9286	> 1000		< 0.5		0,13
	***Streptococcus agalactiae (B)***	7DT1887	< =250		> 4		0,06
	***Streptococcus porcinus***	2EU9285	< =250		< 0.5		0,06
	***Enterococcus faecalis***	2CQ9355	< =500		1	< =0.5/9.5	
	***Enterococcus faecium***	13EU7181	< =500		> 4	> 4/76	
***Gram-negative* Bacilli**	***Acinetobacter baumannii***	7DT2404	< =1	< =2/2		< =1/19	
	***Escherichia coli sauvage***	3DT1938	2	8/2		< =1/19	
	***Escherichia coli BLSE***	2DT2057	2	> 8/2		> 4/76	
	***Enterobacter aerogenes***	07CQ164	< =1	8/2		< =1/19	
	***Enterobacter cloacae***	02EV317	> 4	> 8/2		> 4/76	
	***Citrobacter koseri***	3DT2151	< 1	> 8/2		< 20	
	***Klebsiella pneumoniae***	3DT1823	< =1	< =2/2		< =1/19	
	***Proteus mirabilis***	2DS5461	2	< =2/2		> 1/19	
	***Pseudomonas aeruginosa***	2DT2138	2	> 8/2		4/76	
	***Pseudomonas fluorescence***	5442	4	> 8/2		4/76	
	***Pseudomonas putida***	2DT2140	> 4	> 8/2		> 4/76	
	***Serratia marcescens***	375BR6	4	> 8/2		> 4/76	
	***Salmonella*** sp.	2CG5132	> 4	8/2		> 4/76	
	***Shigella*** sp.	7DS1513	> 4	8/2		> 4/76	
	***Yersinia enterocolitica***	ATCC27729	< =1	8/2		2/38	

#### 3.3.2 Antifungal activity

The EO of *Thymus zygis* species are the most active against candidiasis, followed by the EO of *Thymus vulgaris* and then the two species of *Thymus satureioides,* except *Candida dubliniensis* which is inhibited by a low concentration of *Thymus satureioides* EO from the commune of Azrou (MIC = 18.75 μg/ml), the yeast of *Saccharomyces cerevisiae* was found to be very sensitive to EO of *Thymus vulgaris* species, *Thymus zygis* from Tigrigra and Bensmim communes, with a moderate to low effect towards the two *Thymus satureioides* EO tested. The mold evaluated, *Aspergillus niger* is inhibited with a MIC of 37.5 μg / ml by the EO of *Thymus vulgaris, Thymus zygis* of the communes of Ain Aghbal and Timahdite. Both species of candidiasis; *Candida kyfer* and *Candida krusei* showed some resistance to the EO of the different thymes evaluated, knowing that, even for the standard antifungal “terbinafin”, a higher concentration was needed to inhibit the growth of these two strains in question with a concentration ranging from 25 to 50 μg/ml of terbinafin. In contrast, other fungi are inhibited just by concentrations below 12.5 μg/ml terbinafin (Table 11).

**Table 11:** Results of MIC and MFC (μg/ml) of the essential oils studied and terbinafin.

**Microorganism**	**References**	**TVHA**		**TSAZ**		**TSTA**		**TZTIG**		**TZBE**		**TZAA**		**TZTIM**		**Terbinafin**
		**MIC**	**MFC**	**MIC**	**MFC**	**MIC**	**MFC**	**MIC**	**MFC**	**MIC**	**MFC**	**MIC**	**MFC**	**MIC**	**MFC**	
***Candida albicans***	Ca	600	600	2500	5000	2500	2500	600	1200	300	300	1200	1200	150	300	12,500
***Candida kyfer***	Cky	1200	1200	5000	5000	2500	2500	1200	1200	1200	2500	1200	2500	1200	2500	25,000
***Candida krusei***	Ckr	2500	5000	5000	5000	1200	1200	2500	5000	1200	1200	> 5000	> 5000	2500	2500	50,000
***Candida parapsilosis***	Cpa	150	300	600	600	1200	1200	600	1200	1200	1200	150	300	150	300	6,250
***Candida tropicalis***	Ct	300	300	1200	1200	5000	5000	300	600	600	1200	600	600	600	600	12,500
***Candida dubliniensis***	Cd	600	1200	18,75	37,5	5000	5000	600	600	300	300	600	1200	1200	1200	3,125
***Aspergillus niger***	AspN	37,5	70	300	600	600	600	300	300	300	300	37,5	70	37,5	70	3,125
***Saccharomyces cerevisiae***	Sacc	70	150	600	1200	2500	2500	70	150	70	150	600	1200	300	300	3,125

### 3.4 Correlation of the chemical composition of essential oils (%) with their antimicrobial properties (MIC in mg/ml)

The red and blue colors show the positive and negative correlation respectively, and the correlation coefficient is displayed by the intensity of the color.

**Fig. 6:**
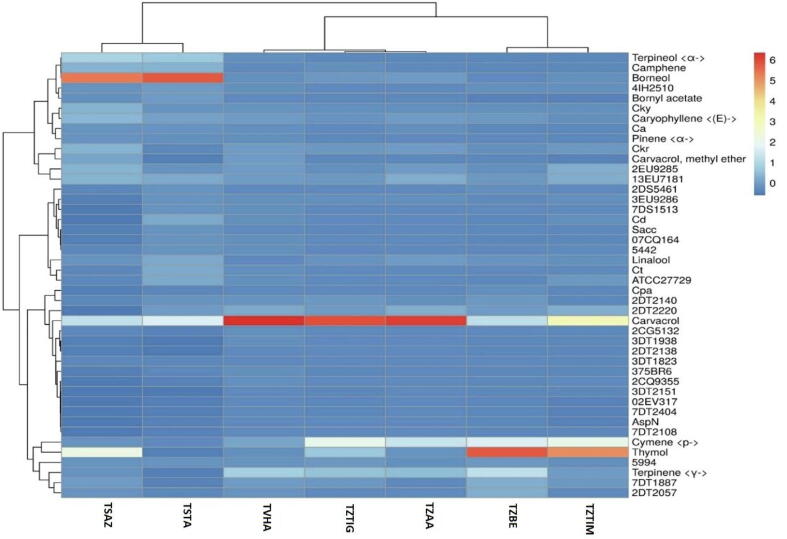
Correlation of the chemical composition of essential oils with their antimicrobial properties measured as inhibition capacity (MIC) (Two-Dimensional Clustered Heatmaps).

The heat map analysis of seven samples of Moroccan thymes based on phenotypic traits is illustrated in Fig. 6. Each small rectangle reflects the percentages of phytochemicals and their antimicrobial property (MIC in mg/ml) of thyme samples. The color represents the normalized value, with red representing the highest value and blue representing the lowest value.

Each line represents the standardized content of different studied characteristics of a species. Each column represents the difference in the standardized results of different varieties in a single specific species.

Fig. 6 shows the correlation between the chemical composition of EO and their effect of inhibiting the growth of the microorganisms tested (MIC). This correlation shows that phenols had the greatest positive effect on antimicrobial properties, followed by terpene compounds and then the other chemical families. Different sensitivity to chemical families was observed for microorganisms tested using inhibition capacity (MIC). In particular, phenols and terpenes were more effective against a greater number of microorganisms such as *Enterobacter cloacae* (02EV317), *Citrobacter koseri* (3DT2151), *Candida parapsilosis* (Cpa), *multidrug-resistant Staphylococcus aureus* (2DT2220), *Pseudomonas aeruginosa* (2DT2138), *Acinetobacter baumannii* (7DT2404) and *Aspergillus niger (*AspN)*.* In addition, the phenol carvacrol present in the species *Thymus vulgaris* (TVHA), *Thymus zygis* of the commune Ain Aghbal (TZAA), and *Thymus zygis* of the commune Tigrigra (TZTIG) has a strong positive correlation about the inhibition of the growth of gram-negative bacteria as well as *Candida parapsilosis* (Cpa), *Aspergillus niger* (AspN) and *Candida tropicalis* (Ct). An example of a weak correlation is that of carvacrol with *Candida krusei* (Ckr) and *Candida kyfer* (Cky). In addition, it is known that genetic makeup and environmental conditions influence the yield and composition of the volatile oil produced by thyme plants. Correlations between chemotypes polymorphism, sexual polymorphism, and environment have been detected (Gouyon et al. 1986). The antifungal and antibacterial activity of the essential oil of the genus *Thymus* has been demonstrated by several researchers (Vinciguerra et al., 2019, El-Shemy, 2017, Delgado-Adámez et al., 2017, El Hachlafi et al., 2021). Most studies report the action of essential oils against pathogens. This work agrees that essential oils are relatively more active against Gram-positive bacteria than Gram-negatives (Mutlu-Ingok et al. 2021). Deans et al. (Deans et Ritchie 1987) observed that the sensitivity of Gram positives and Gram-negative bacteria to volatile vegetable oils influenced growth inhibition. However, some oils have appeared to be more active concerning the Gram reaction, exerting greater inhibitory activity against Gram-positive bacteria; it has often been reported that Gram-negative bacteria are more resistant to essential oils present in plants (Smith-Palmer, Stewart, et Fyfe 1998). The structure of the cell wall of Gram-negative bacteria consists mainly of Lipopolysaccharides (LPS). This constituent prevents the accumulation of oils on the cell membrane (Bezić et al., 2003).

The results obtained in our study showed that Gram-negative bacteria were more sensitive to the essential oils of thymes. On the other hand, these EO showed a certain selectivity towards Gram-positive bacteria. Moreover, the biological activity of essential oils depends on their chemical composition, which is determined by the genotype and influenced by environmental and agronomic conditions (Marotti et al. 1992). Much of the antimicrobial activity of *Thymus* essential oils appears to be associated with phenolic and terpene compounds (carvacrol, thymol, borneol, p-cymene, and γ-Terpinene) (Davidson and Naidu, 2000, Skočibušić et al., 2006, Cosentino et al., 1999).

In this research, the antimicrobial activity of oils may be due to carvacrol, thymol, borneol, p-cymene and γ-terpinene. According to literature reviews, the above-mentioned bioactive molecules have been found to have significant antimicrobial activities (Aghraz et al., 2017, Vardar-Ünlü et al., 2003). Furthermore, phenols, non-aromatic alcohols and monoterpene hydrocarbons of the following types: carvacrol, thymol, borneol, p-cymene and γ-terpinene are well-known chemicals with antimicrobial potential (Dorman et Deans 2000).

The antimicrobial activity of thyme essential oils is related to its phenol-like components, non-aromatic alcohols, and monoterpene hydrocarbons (Fig. 3) since there is a relationship between the chemical structures of the most abundant oils and their antimicrobial activities. Although the mechanism of action of phenols and terpenes is not fully understood, it is thought to involve the rupture of the membrane by lipophilic compounds (Cowan 1999). Essential oils containing phenols and terpenes possess remarkable antimicrobial activity (Dorman et Deans 2000), which is consistent with our current study. The synergistic effects of these active chemicals with other constituents of essential oils should be considered for antimicrobial activity.

## 4 Conclusion

The essential oils of Moroccan thymes contain as main components; carvacrol in the EO of Thymus vulgaris from El Hammam of the province of Mrirt, the borneol/carvacrol chemotype for the two Thymus satureioides from the commune of Tata and the commune of Tigrigra, as well as a dominance of the carvacrol/thymol/p-cymene and γ-terpinene chemotype for Thymus zygis subsp. Gracilis. The samples of the evaluated thymes have a strong antimicrobial power with a bactericidal and fungicidal effect against the evaluated microbial strains. Pharmaceutical, food and cosmetic industries need ecological alternatives to drug molecules to treat infectious diseases. Thus, these EOs could be a potential source of alternative antimicrobial agents and could play an important role in the discovery of new products against a wide range of pathogenic microorganisms in the near future.

## Declaration of Competing Interest

The authors declare that they have no known competing financial interests or personal relationships that could have appeared to influence the work reported in this paper.
